# Physiological and Psychological Predictors of Functional Performance Related to Injury Risk in Female Athletes: A Cross-Sectional Study

**DOI:** 10.3390/healthcare14020174

**Published:** 2026-01-09

**Authors:** Monira I. Aldhahi, Hadeel R. Bakhsh, Bodor H. Bin sheeha, Mohanad S. Aljabiri, Rehab Alhasani

**Affiliations:** 1 Department of Rehabilitation Sciences, College of Health and Rehabilitation Sciences, Princess Nourah bint Abdulrahman University, P.O. Box 84428, Riyadh 11671, Saudi Arabia; hrbakhsh@pnu.edu.sa (H.R.B.); bhbinsheeha@pnu.edu.sa (B.H.B.s.); rsalhasani@pnu.edu.sa (R.A.); 2 Lifestyle and Health Research, Health Science Research Center, Princess Nourah bint Abdulrahman University, Riyadh 11671, Saudi Arabia; msaljabiri@pnu.edu.sa

**Keywords:** exercise performance, fitness, risk of injury, self-efficacy, strength, sports

## Abstract

**Highlights:**

**What are the main findings?**

- This study investigated the combined physiological and psychological predictors of lower-extremity injury risk in female athletes.
- Single-Leg Hop distance (SLH), aerobic capacity (VO_2peak_), and self-efficacy emerged as significant predictors of functional performance on the Lower Extremity Functional Test (LEFT).

**What are the implications of the main findings?**

- Higher aerobic fitness, SLH, and self-efficacy were associated with better LEFT performance, reflecting superior functional performance on a test commonly used as a proxy for lower-extremity injury risk.
- The findings support integrating assessments of SLH, VO_2peak_, and self-efficacy as efficient screening tools for targeted injury prevention strategies.

**Abstract:**

**Background and Objectives:** Lower-extremity injuries are common among female athletes; however, their multifactorial predictors remain insufficiently understood. Given the interplay between physiological and psychological readiness in athletic performance, identifying the factors that influence lower limb performance is crucial for effective injury prevention. This study aimed to evaluate the predictive effects of physiological (VO_2_peak, anaerobic power, agility, and isokinetic strength) and psychological (resilience and self-efficacy) variables on functional performance related to risk of injury. **Materials and Methods:** This cross-sectional study included 60 athletes with a mean age of 24.5 ± 6.90 years and mean body mass index of 23.12 ± 3.6 kg/m^2^ (range: 16–30 kg/m^2^). The testing protocol included anthropometric measurements, the Lower Extremity Functional Test (LEFT), Wingate anaerobic cycling test, assessments of aerobic capacity, isokinetic muscle strength, and jumping performance (Single-Leg Hop [SLH] and Standing Long Jump [SLJ] tests). Psychological assessments included the General Self-Efficacy Scale (GSES) and a resilience questionnaire. A hierarchical regression analysis was performed. **Results:** The participants trained 5 ± 2 days per week and had 42 ± 39 months of sports experience. The mean VO_2peak_ was 40.82 ± 5.8 mL·kg^−1^·min^−1^, relative anaerobic peak power was 7.53 ± 1.92 W/kg, and fatigue index was 60.63 ± 15.41%. The mean isokinetic knee extension and flexion torque were 184.55 ± 44.60 N·m and 95.08 ± 21.44 N·m, respectively, with a flexion-to-extension ratio of 53.5%. The mean LEFT completion time was 160 ± 22 s. The participants demonstrated moderate resilience (BRS = 21 ± 4) and good self-efficacy (GSES = 33 ± 7.5). Among the psychological variables, GSES exhibited a modest negative correlation with LEFT (r = −0.28, *p* = 0.02). No significant associations were found between LEFT and psychological resilience. Longer LEFT completion times were associated with lower VO_2peak_, mean power, and jump distance (*p* < 0.01). In the final model (R^2^ = 0.58, *p* = 0.02), SLH (β = −0.54), VO_2peak_ (β = −10.32), and GSES (β = −0.70) were the strongest independent predictors of LEFT performance. **Conclusions:** SLH distance, VO_2peak_, and general self-efficacy are key predictors of functional performance on the LEFT among female athletes. These factors may serve as practical indicators for identifying athletes who could benefit from targeted injury prevention programs.

## 1. Introduction

The global landscape of competitive sports is witnessing an unprecedented surge in female participation, which is prominently reflected in Saudi Arabia’s evolving sports culture. This transformative shift aligns seamlessly with the Kingdom’s Vision 2030, which emphasizes empowering women across diverse sectors, including a dedicated focus on increasing female participation in sports. Vision 2030 provides comprehensive support for female athletes to compete at various levels [1,2]. Key milestones already achieved include the establishment of women’s sports leagues and teams across various disciplines [1,3]. While the increasing inclusion of women in sports, particularly those demanding significant physical and psychological exertion, marks a crucial advancement towards gender equity, it also necessitates the careful consideration of the concurrent rise in sports-related injuries. The risk of injury poses potential ramifications for both athletes’ well-being and the overall healthcare infrastructure [4].

The increasing participation of women in competitive sports represents a significant advancement in gender equity and public health promotion. However, this expansion has been accompanied by a parallel rise in sports-related injuries among female athletes, with lower-extremity injuries being the most prevalent [5,6,7]. Recent local surveillance has underscored that during Saudi Arabia’s first national women’s basketball, 15.9% of players sustained injuries, predominantly lower-limb injuries, over one season [7,8]. A university-based survey in Riyadh found that 62.2% of female athletes had a history of sports-related injury [5]. Evidence from both local and international studies indicates that female athletes experience a higher incidence of sports injuries compared with their male counterparts, leading to prolonged recovery periods, time away from sport, and increased healthcare burden [9,10]. From a physiological standpoint, women’s physiological systems may not be fully adapted to the rigorous demands of sustained athletic activity [11], underscoring the critical importance of injury-prevention programs. These findings highlight the importance of developing targeted injury-prevention strategies that address the unique physiological and psychological characteristics of female athletes.

The etiology of sports injuries is multifactorial, involving an interplay between intrinsic and extrinsic factors [7]. Among intrinsic factors, various physiological attributes have been investigated as potential determinants of injury risk [7]. In theory, maximizing athletes’ physical fitness, including muscular strength, anaerobic power, aerobic capacity, balance, and agility, is believed to help limit the risk of injury. For instance, greater pre-season aerobic fitness has been associated with a reduced risk of in-season injury among female soccer players [12]. In addition, functional agility tests have shown predictive value; slower times on the Lower Extremity Functional Test (LEFT), a shuttle run agility test, correlate with higher susceptibility to subsequent lower extremity injuries [13]. Functional performance testing has emerged as a practical method for identifying athletes at an increased risk of injury. The LEFT is a multidirectional agility-based assessment that integrates strength, power, balance, coordination, and aerobic capacity. Previous studies have demonstrated that prolonged LEFT completion times are associated with a higher likelihood of subsequent lower-extremity injuries, supporting its use as a surrogate indicator of injury risk in athletic populations [13,14]. Although LEFT performance does not directly measure injury incidence, it provides a valid and clinically feasible proxy for identifying athletes with functional deficits linked to elevated injury susceptibility, particularly in cross-sectional and screening-based designs [15].

Beyond physical attributes, psychological factors play an essential role in injury risk and recovery. Psychological resilience, coping strategies, and confidence have been shown to influence rehabilitation adherence, return-to-sport decisions, and performance under pressure [16,17]. Athletes with higher psychological readiness often demonstrate better training engagement and more favorable recovery trajectories following injuries [18]. Self-efficacy, defined as an individual’s belief in their ability to perform the required actions successfully, is a particularly relevant psychological construct in athletic populations [19]. Higher self-efficacy is associated with improved performance, greater adherence to rehabilitation programs, and enhanced ability to manage physical and psychological challenges during training and competition [19]. Conversely, insufficient psychological readiness may increase vulnerability to injury and reinjury, highlighting the need for consideration in injury risk assessment [16,20]. Despite the growing recognition of the importance of psychosocial factors, injury risk evaluation and return-to-sport decisions continue to rely predominantly on physical healing and performance measures. This underscores the need for more comprehensive approaches that integrate both physiological and psychological components when assessing injury risk and functional readiness in female athletes.

Sports-related injuries among female athletes are influenced by complex interactions among physiological, biomechanical, and psychological factors [21]. Recognizing these interrelated factors highlights the importance of adopting a multifactorial and sex-specific approach when evaluating injury risk and functional performance in female athletic populations. It is crucial to address the relative lack of research explicitly focused on female athletes, as much of the existing knowledge has been extrapolated from studies primarily conducted on male populations. Understanding associated risk factors in women is essential, as sex-specific physiological, biomechanical, and psychological differences may influence injury susceptibility [11]. By exploring the interaction between physiological predispositions and psychological factors, a more holistic approach to injury prevention and management can be achieved. Such an approach is vital for safeguarding the health, performance, and long-term athletic participation of female athletes in the Kingdom and elsewhere.

The current gap in female-specific research poses a significant challenge for designing targeted, sustainable injury prevention programs. In light of these considerations, this study aimed to comprehensively examine the physiological and psychological dimensions of sports-related injuries among female athletes. Specifically, this study sought to assess the predictive effects of selected physiological and psychological factors on injury risk. On a physiological level, markers such as isokinetic muscle strength, anaerobic power, fatigue rate, and oxygen consumption were examined. Concurrently, the psychological domain was evaluated using validated measures of self-efficacy (General Self-Efficacy Scale) and resilience (Resilience Scale). Based on existing evidence, we hypothesized that specific physiological markers (isokinetic muscle strength, anaerobic power, fatigue rate, and oxygen consumption) and psychological factors (self-efficacy and resilience) would significantly predict the risk of sports-related injuries in female athletes. These findings may support the development of evidence-based, personalized prevention strategies tailored to the unique needs of female athletes.

## 2. Materials and Methods

### 2.1. Study Design and Participants

This cross-sectional study was conducted between March and August 2025. The trial was registered with ClinicalTrials.gov (ID: NCT07026383) and conducted in accordance with the STROBE Statement guidelines for reporting observational studies [22]. This study was conducted at the Lifestyle and Health Research Laboratory. All assessments were performed by a qualified multidisciplinary team comprising licensed physical therapists and certified clinical exercise physiologists following standardized protocols.

Female athletes aged 18–35 years who were actively participating in any sport in Saudi Arabia were eligible for inclusion in the study. Participants were required to have at least one year of continuous sport-specific experience, to have engaged in regular training for at least three sessions per week, and to have been actively involved in organized training and competitive events at the collegiate or club level. Participants were excluded if they presented with conditions that could confound functional performance outcomes or compromise safety. Specifically, individuals with a musculoskeletal injury within the preceding six months affecting the lower/upper extremities or spine were excluded to avoid residual effects on movement patterns, neuromuscular control, and performance capacity. Pregnant participants or athletes with chronic medical conditions that could affect exercise tolerance or neuromuscular function, such as cardiovascular, respiratory, neurological, or metabolic disorders, were also excluded, including those with uncontrolled diabetes or hypertension. Participants using medications known to alter cardiovascular or neuromuscular responses (e.g., beta-blockers or systemic corticosteroids) were also excluded. To ensure a homogeneous athletic profile, individuals who did not meet the minimum training exposure of at least three organized training sessions per week were excluded.

A priori sample size calculation was performed to estimate the sample size. The sample size was determined using G*Power software (version 3.1.9.6, Heinrich Heine University Düsseldorf, Düsseldorf, Germany) for multiple linear regression analysis. With an alpha level of 0.05, 80% power, and seven independent predictors, a moderate effect size (f^2^ = 0.35) yielded a required sample of approximately 60 female athletes. The invitation to participate in the study was widely disseminated through official channels, including the Saudi Sports Federations and the Ministry of Sports. Participant recruitment employed a non-probability, convenience sampling method. Enrollment was open to all eligible female collegiate athletes who responded to the invitation within the data collection timeframe.

This study was conducted in accordance with the principles of the Declaration of Helsinki. Ethical approval was obtained from the Institutional Review Board of Princess Nourah bint Abdulrahman University (24-0938). All participants were informed of the study procedures, potential risks, and benefits prior to participation. Written informed consent was obtained from all eligible participants prior to their enrollment in this study. Participation was voluntary, and the confidentiality of all participant data was maintained throughout the study.

### 2.2. Study Procedures

**Figure 1:**
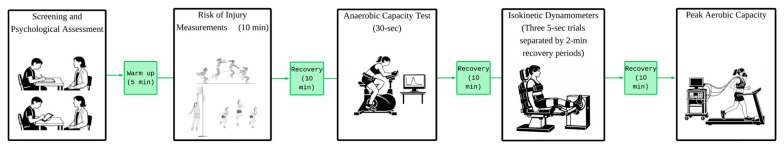
Study protocol sequence of assessments and recovery between testing stations.

The study protocol is illustrated in Figure 1. Participants were instructed to follow standardization requirements, including refraining from intense physical activity for 24 h and avoiding caffeine or other stimulants for at least 12 h prior to testing. After providing informed consent, participants underwent body composition testing, after which they were provided with a light snack and water to replenish energy and ensure hydration. Before testing, all participants completed a standardized 5 min dynamic warm-up to prepare their musculoskeletal and cardiovascular systems and reduce the risk of injury. Participants completed the assessments in the following order in a single day: screening and psychological assessment (5 min), warm-up (10 min), risk-of-injury measurements (10 min), followed by a recovery period. Muscular assessment and anaerobic capacity testing were performed using three 5 s trials separated by 2 min recovery periods, followed by a 10 min recovery period. Finally, the peak aerobic capacity was assessed. Environmental conditions were controlled with a laboratory temperature maintained at 18–22 °C, a relative humidity of 40–60%, and adequate ventilation [23].

#### 2.2.1. Anthropometric and Body Composition

Anthropometric and body composition measurements were obtained while the participants wore minimal clothing to ensure accuracy. Height and weight were measured using an electronic measuring station (Seca 285, Seca, Hamburg, Germany), with height recorded to the nearest 0.5 cm and weight recorded to the nearest 0.1 kg. Body mass index (BMI) was calculated by dividing weight in kilograms by height in meters squared (kg/m^2^).

Whole-body and segmental body composition, including fat mass (FM) and skeletal muscle mass, was assessed using Bioelectrical Impedance Analysis [24]. Measurements were conducted following an overnight fasting period of at least six hours to minimize variability. A multi-frequency BIA device (mBCA 525; Seca, Hamburg, Germany) was used, applying a consistent high-frequency electrical current of 100 mA at 50 kHz to ensure precise and reproducible results.

#### 2.2.2. Muscular Assessment and Anaerobic Capacity

Knee extensor and flexor muscle strength were evaluated using a Humac NORM dynamometer (CSMi, Stoughton, WI, USA). The participants were positioned in the dynamometer chair with the hip flexed at approximately 110°, ensuring proper anatomical alignment. The posterior aspect of the knee was aligned with the seat edge, which was adjusted to 60° from anatomical zero (180°), a position previously shown to optimize maximal isometric force production [25]. Each participant performed one familiarization trial, followed by three maximal voluntary contractions. Peak torque was recorded using the Humac Norm Isokinetic Software System, which provided real-time visual feedback to encourage maximal efforts.

Anaerobic performance was assessed using the Wingate Anaerobic Test (WAnT) on a cycle ergometer (Monark 894E, Vansbro, Sweden), which is a reliable measure of anaerobic power [26]. The seat height was individually adjusted to ensure proper biomechanics and the participant’s comfort. Prior to the test, participants completed a standardized two-minute warm-up at 60 revolutions per minute (rpm) without resistance to optimize their readiness. The test consisted of a 30 s all-out sprint against a resistance set at 7.5% of the participant’s body mass [26]. The performance outcomes included peak power (PP) in watts, average power (AP) in watts, and Fatigue Index (FI) in percentage. Peak power was defined as the highest mechanical power output achieved during a 30 s sprint. The FI was derived using the following formula [27]:

FI (%) = [1 − (Minimum power in W·kg^−1^ ÷ Peak power in W·kg^−1^) × 100.

#### 2.2.3. Peak Aerobic Capacity

**Figure 2:**
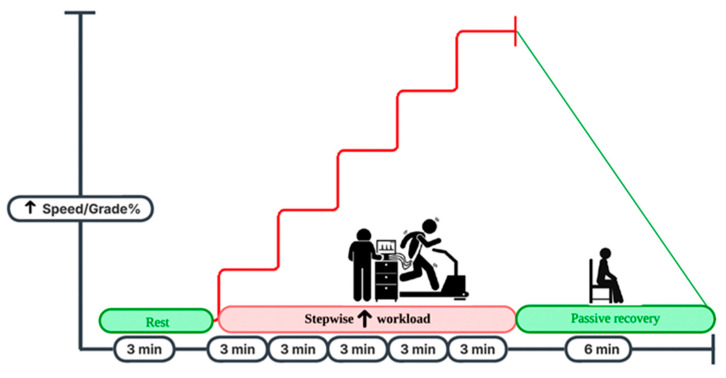
Illustration of the Bruce treadmill protocol.

Peak Oxygen consumption (VO_2peak_), a widely recognized surrogate for cardiopulmonary fitness, was quantitatively assessed using a breath-by-breath cardiopulmonary function analyzer (MetaLyzer 3B-R3, Cortex, Germany). After a three-minute seated recovery period, the participants were fitted with a facemask and completed the modified Bruce Protocol to volitional exhaustion (Figure 2) [28]. The incremental treadmill protocol consisted of three-minute stages, beginning at 2.74 km/h with a 10% incline, with subsequent increases in both the gradient and speed according to the original Bruce guidelines [28]. Oxygen uptake (VO_2_), carbon dioxide production (VCO_2_), respiratory exchange ratio (RER), and ventilation parameters were measured continuously throughout the test. Heart rate (HR) was monitored using a short-range telemetry monitor (Polar H7, Kempele, Finland). Participants’ ratings of perceived exertion (RPE) were collected every 2 min using the Borg scale. The test was concluded either when the participants reached volitional exhaustion or when at least two established physiological markers indicative of maximal aerobic capacity were observed. These included a respiratory exchange ratio (RER) equal to or greater than 1.15, attainment of 90% or more of the age-predicted maximal heart rate, a plateau in oxygen uptake (VO_2_) despite increasing workload—defined as an increase of less than 150 mL/min—and a rating of perceived exertion of 18 or higher on the Borg 6–20 scale [29].

#### 2.2.4. Risk of Injury Measurements

A battery of standing long jump tests and lower extremity functional performance protocols [30] was used. The LEFT includes eight agility-based drills executed consecutively on a diamond-shaped course, with no rest intervals between tasks [15,31]. The test consisted of two repetitions of each of the following movements: forward sprint, backward run, lateral shuffle, carioca (grapevine), figure-eight run, 45° directional cuts, 90° directional cuts, and a counter movement vertical jump. It is recognized for its ability to predict injury risk in female collegiate athletes, with a recommended performance time of 135 s and a range of 120–150 s [31,32]. Additionally, athletes with slower LEFT scores (≥118 s) experienced significantly higher injury rates than those with faster performance (≤117 s) [31]. Intraclass correlation coefficients (ICCs) was high (ICC = 0.97) [33], and the test showed significant accuracy discriminating between injured and uninjured players (AUC 0.908, 95% CI 1.126–1.336, *p* = 0.001) [13].

In addition, the Single-Leg Hop (SLH) and Standing Long Jump (SLJ) Distance Tests were performed, which is a protocol previously validated for predicting injury risk [34,35,36]. SLH for Distance–The participant stands on one leg and jumps forward as far as possible, landing on the same leg without losing balance. The best distance from multiple trials is recorded. In the SLJ test, the participant stands with both feet behind a starting line, swings their arms, and jumps forward explosively, landing on both feet. The distance from the starting line to the heel closest to it is measured. Both the SLH for Distance and SLJ can be used as part of a battery of tests to help predict injury risk, especially in sports and rehabilitation contexts.

#### 2.2.5. Countermovement Jump (CMJ) Assessment

Vertical jump height was assessed using a Vertec device (Sports Imports, Hilliard, OH, USA), which consists of 50 plastic swivel vanes, each spaced 1.0 cm apart, mounted on a telescopic metal pole. Prior to testing, the device was individually adjusted so that the lowest vane was aligned with each participant’s standing reach height. The participants were instructed to perform three maximal vertical jumps, displacing the highest possible vane using an overhead arm swing with their dominant hand. All jumps were initiated from a standardized position, with the participants standing 10 cm away from the device. The final jump height was calculated by subtracting the standing reach from the maximal jump reach [37]. Based on the jumping performance, the peak anaerobic power (PAP) equation from previous studies was used: Squat jump Peak Power (W) = 60.7 × height (cm) + 45.3 × body mass (kg) − 2055 [38]. The test–retest reliability was good (ICC = 0.97) [39]. The structural validity of CMJ has been evaluated using factor analysis. The CMJ demonstrated the highest loading on the explosive power factor (r = 0.87), confirming factorial validity [40].

#### 2.2.6. Psychological Assessment

The validated Arabic version of the General Self-Efficacy Scale (GSES) was used in this study, which minimizes potential differences in interpretation due to language or cultural context [41]. This version showed strong psychometric properties, with a unidimensional structure confirmed by CFA (RMSEA = 0.07, CFI = 1.00, TLI = 0.99), supporting its reliability and validity [41]. This validated instrument is widely employed to assess an individual’s confidence in their ability to navigate challenges, adapt to changing demands, and achieve desired outcomes in both everyday and high-stress situations. The GSES comprises 10 unidimensional self-report items (e.g., “I can always manage to solve difficult problems if I try hard enough”), each rated on a four-point Likert scale. The cumulative score ranges from 10 to 40, with higher scores reflecting a stronger conviction in one’s personal competence to manage adversity and exert effective control over one’s life circumstances.

The Brief Resilience Scale (BRS) was used in this study to evaluate individuals’ capacity to recover from stress and adapt positively to health-related challenges [42,43]. The validated Arabic version of the BRS was used in this study, which has been widely employed and has demonstrated strong reliability and validity for assessing resilience among the Arabic-speaking Saudi population. (α = 0.98 and ICC = 0.88) [43]. The BRS specifically targets the ability to “bounce back” from adversity, thereby capturing a fundamental component of psychological resilience. This unidimensional, self-administered instrument consists of six items, each rated on a five-point Likert scale ranging from 1 (Strongly Disagree) to 5 (Strongly Agree), resulting in a total score range of 6–30. Higher scores indicate greater resilience. The scale has demonstrated strong psychometric validity and reliability across diverse populations, making it a robust and efficient tool for assessing resilience in health-related research [43].

### 2.3. Statistical Analysis

Data were analyzed using Stata version 17 (StataCorp LLC, College Station, TX, USA). Continuous variables are presented as mean ± standard deviation (SD), while categorical variables are summarized as frequency (N) and percentages (%). Data distribution normality was assessed using the Shapiro–Wilk test and visually inspected using histograms. Pearson’s correlation coefficient (r) and univariate linear regression were used to determine the associations between LEFT and various physiological and psychological parameters. The strength of correlations was interpreted as small (r = 0.10–0.29), moderate (r = 0.30–0.49), and large (r ≥ 0.5) [44]. Physiological variables that showed significant associations in the univariate analyses were entered into the first block of the hierarchical regression model. Psychological variables were subsequently added in the second block to assess their incremental contribution beyond physiological determinants. Changes in the explained variance (ΔR^2^) were used to evaluate the added predictive value of psychological factors. Statistical significance was set at *p*-value < 0.05. The figure illustrating the correlations was generated using GraphPad Prism (version 10, GraphPad Software, San Diego, CA, USA).

## 3. Results

The physical and training characteristics of the participants are shown in Table 1. A total of 60 female participants, with an average age of 24.50 ± 6.90 years (range: 19–45), were included in the study. The body mass index (BMI) ranged from 16 kg/m^2^ to 30 kg/m^2^. Participants demonstrated a moderate training load, with regular weekly training and several years of sports experience (range: 2–13 years). A range of sports disciplines was represented, with volleyball and rugby being the most common, indicating diverse training demands.

The descriptive data of anaerobic performance are presented in Table 2. The participants demonstrated a moderate-to-high level of anaerobic capacity in comparison to normative data, with mean absolute peak power values reaching 448.06 ± 123.23 Watts (W), and relative peak power averaging 7.53 ± 1.92 W/kg. The mean fatigue index was 60.63%, indicating a rapid decline in power output. Muscular strength values, including peak torque for knee extension and flexion (184.55 ± 44.60 N·m and 95.08 ± 21.44 N·m, respectively), reflected a balanced profile, as shown by an average flexion-to-extension ratio (F:E) of 53.5%.

Additionally, aerobic responses, as reflected by VO_2peak_ and peak HR, indicated that the players demonstrated a moderate-to-high level of cardiovascular fitness. The mean VO_2peak_ was 40.82 ± 5.8 mL·kg^−1^·min^−1^, which falls within the fair to good category for cardiorespiratory fitness [45]. The peak heart rate of 180 bpm and an average RER of 1.10 indicated that the participants reached maximal effort during the test. The ventilatory equivalents for oxygen (VE/VO_2_) and carbon dioxide (VE/VCO_2_) were both approximately 32, aligning with normative values and indicating efficient gas exchange without evidence of ventilatory inefficiency.

Table 3 presents several functional and injury risk-related parameters of the participants. The average completion time for the LEFT was relatively prolonged compared to the normative (<135 s) [31,32]. The performance on the SLH test was symmetrical on average, with a side-to-side difference of approximately 7 cm, which was within the acceptable threshold. While asymmetries greater than 10% are generally associated with a higher injury risk, the observed difference remained within acceptable margins. A reasonably good level of horizontal power relative to stature was observed in the SLJ, averaging 156.61 cm, approximately 98% of the participant’s height. Similarly, the CMJ height of approximately 33 cm corresponded to a mean peak anaerobic power output of 3066.90 W (ranging from 1762.8 to 4598.5 W), which is higher than the reported average peak power output for females (2993.7 ± 542.9 W). Psychologically, the participants scored moderately on both the brief resilience scale and the GSES, yielding mean scores of 21 and 33, respectively.

**Figure 3:**
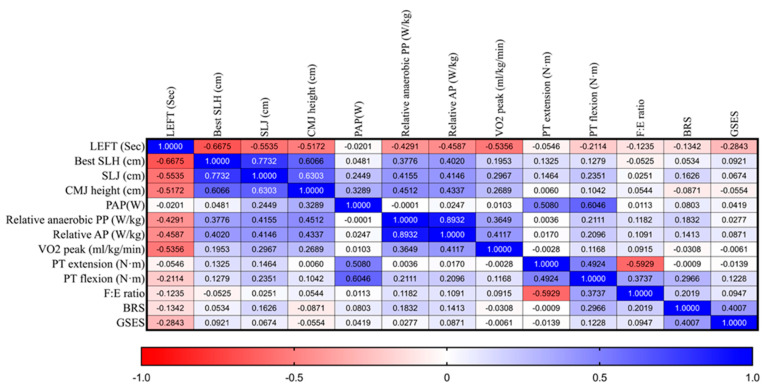
Heatmap Matrix of Pearson Correlation coefficient Between LEFT mechanical outcome of anaerobic power test and aerobic fitness. The colors indicate the direction and strength of the effects.

Figure 3 and Table 4 show the correlation heatmap, which illustrates the correlation matrix and highlights significant relationships between LEFT and various physiological and psychological profiles. Red indicates a close negative correlation, while blue indicates a positive correlation in the data. Notably, LEFT scores were significantly and inversely correlated with multiple performance measures, suggesting that poorer LEFT performance (longer completion time) was strongly negatively correlated with SLH (r = −0.66, *p* < 0.001), SLJ (r = −0.55, *p* < 0.001), and countermovement jump height (r = −0.51, *p* < 0.001) (Table 4).

Additionally, LEFT performance was significantly negatively associated with relative anaerobic peak power (r = −0.43, *p* = 0.0005), mean power (r = −0.44, *p* = 0.0004), and VO_2peak_ (r = −0.53, *p* < 0.001) in WAnT. Among the psychological variables, GSES exhibited a modest negative correlation with LEFT (r = −0.28, *p* = 0.02). No significant associations were found between LEFT and muscular strength (PT extension/flexion) or psychological resilience.

Univariate linear regression analysis (Table 5) revealed that LEFT, as a proxy for the risk of injury, was significantly predicted by multiple mechanical and psychological variables. The strongest predictor was the SLH distance, with a standardized beta (β) of −0.66 (*p* < 0.001), explaining 43% of the variance. Other significant predictors included SLJ (β = −0.55, *p* < 0.001), CMJ height (β = −0.51, *p* < 0.001), and VO_2peak_ (β = −0.53, *p* < 0.001). Both peak power (PP) and mean power (MP) also significantly predicted LEFT (*p* < 0.01). Additionally, general self-efficacy was a modest but significant predictor (β = −0.28, *p* = 0.02).

Table 6 presents the results of the hierarchical regression analysis with the Test LEFT as the dependent variable. In Model 1, physiological variables were entered, and the model explained 53% of the variance in LEFT performance (R^2^ = 0.53, *p* < 0.001). Within this model, SLH (β = −0.58, *p* < 0.001) and VO_2peak_ (β = −10.84, *p* < 0.001) emerged as significant independent predictors of LEFT performance. In contrast, SLJ, CMJ, peak power (PP), and average power (AP) were not significant contributors. In Model 2, the psychological variable GSES was added to the model, resulting in a significant increase in explained variance (ΔR^2^ = 0.05), with the final model accounting for 59% of the variance in LEFT performance (R^2^ = 0.59). In this model, SLH, VO_2peak_, and GSES remained significant predictors of LEFT performance (F = 6.62, *p* = 0.02).

## 4. Discussion

This study provides empirical support for the role of physiological and psychological factors in predicting injury risk in female athletes. The average LEFT completion time in our cohort exceeded the commonly reported normative cutoffs for athletic performance [31,32], suggesting a higher baseline functional risk of lower-extremity injury. This is consistent with previous evidence linking slower LEFT performance to an increased injury incidence. Our findings demonstrate that functional performance is best explained by a combination of task-specific physical capacity and psychological readiness. Specifically, SLH distance, VO_2peak,_ and general self-efficacy emerged as significant independent predictors of LEFT performance. These results emphasize the importance of horizontal power, aerobic fitness, and confidence in performing demanding lower extremity tasks. In contrast, isokinetic muscle strength, anaerobic power, and psychological resilience did not significantly contribute to the multivariate model. This suggests that absolute strength or generalized psychological traits may be less sensitive predictors of complex functional tasks, such as LEFT performance. It is also possible that strength levels within this cohort were sufficient and therefore not performance-limiting, or that other neuromuscular characteristics (e.g., rate of force development) played a more prominent role. Overall, these findings reinforce the multifactorial nature of injury-related functional performance in female athletes and highlight the need to prioritize task-specific physical measures and psychological self-efficacy when assessing injury risk and designing preventive strategies for female athletes.

The participants demonstrated moderate-to-high levels of anaerobic capacity and aerobic fitness, with a mean relative peak power of 7.53 ± 1.92 W/kg. Although direct normative comparisons for Wingate peak power are sport-specific, these values contribute to the growing body of literature characterizing power output in female athletes [46,47]. However, the observed fatigue index of 60.63% indicates a rapid decline in power output, which has been linked to increased susceptibility to injury and decreased athletic performance, particularly during prolonged or repeated exercise [48]. Aerobically, the participants’ mean VO_2peak_ aligned with the “fair-to-good” fitness category for females in this age group [49]. This is consistent with the findings in similar athletic cohorts; for example, a study on elite female futsal players reported a comparable mean VO_2peak_ of 40.0 ± 5.0 mL/kg/min [50]. Regarding muscular strength, the average flexion-to-extension (F:E) ratio generally reflects balanced muscular capability. This ratio, where the hamstrings are approximately 50–60% as strong as the quadriceps, is considered within the healthy range for female athletes [51]. Despite these healthy strength profiles, isokinetic muscle strength did not significantly contribute to the variance in injury risk measures in the multivariate model of our study, suggesting that while foundational, this specific measure of strength might not be the primary determinant of functional performance in this cohort, or that other aspects of strength are more critical predictors.

In terms of functional performance and injury risk indicators, the average LEFT completion time of 160 ± 22 s in our sample was notably prolonged compared to the normative cutoff of <135 s recommended for female collegiate athletes [32]. Furthermore, while SLH side-to-side differences of 6.96 cm were within acceptable asymmetry thresholds (typically <10) [52], the overall SLH performance, along with standing long jump (156.61 cm) and countermovement jump height (33.08 cm), and peak anaerobic power (3066.90 ± 638.53 W) provided a comprehensive picture of the athletes’ explosive power and functional capabilities [53]. These horizontal and vertical jump metrics are widely recognized as indicators of lower extremity function and performance potential [54]. From a psychological standpoint, participants scored moderately on both the Brief Resilience Scale (21 ± 4) and the GSES (33 ± 7.5). These scores indicate a reasonable level of psychological resilience and self-efficacy, which are essential attributes in athletic populations and influence performance, coping with adversity, and adherence to training and rehabilitation [55,56]. Psychological factors, such as self-efficacy, resilience, and fear of re-injury, also play critical roles in both injury occurrence and recovery [18,57].

From a physiological perspective, studies have shown that variability in individual physiology may contribute to injury incidence [16,58]. From a physiological perspective, studies have shown that variability in individual physiology may contribute to the incidence of injury [59]. The correlation analyses further highlighted the interplay between physiological, functional, and psychological domains. In this study, Poorer LEFT performance (longer times) showed strong negative correlations with SLH, SLJ, and CMJ, reinforcing the association between lower limb power and agility-based tasks. This finding aligns with a previous study that found the SLH test has potential value when integrated into a comprehensive battery of functional performance assessments for female collegiate athletes [34]. However, its utility as an independent screening tool for identifying individuals at elevated injury risk remains uncertain and lacks conclusive evidence [34].

Additionally, significant associations with VO_2peak_ and anaerobic power indices (PP and MP) underscore the multifactorial nature of functional performance in this population. Previous studies align with these findings; for instance, research has emphasized the critical role of anaerobic capacity [60,61] and aerobic conditioning [29,62] in injury prevention among athletes, particularly in mitigating lower-extremity injury risk. The modest yet significant negative correlation with the GSES aligns with the literature, suggesting that psychological readiness contributes to physical task efficiency [19].

The muscular strength profiles of the participants, particularly the average flexion-to-extension (F:E) ratio, did not significantly contribute to the variance in injury risk measures in this study. These findings align with previous literature, which has highlighted the limited predictive value of isokinetic measures in relation to anterior cruciate ligament (ACL) injury risk [63,64]. However, Isokinetic testing remains the gold standard for quantifying muscle strength deficits after ACL injury or reconstruction, aiding in rehabilitation [65]. Therefore, combining isokinetic measures with functional tests (e.g., hop tests) and patient-reported outcomes improves the ability to identify high-risk individuals; however, no single isokinetic parameter is a strong standalone predictor.

In the present study, the univariate linear regression analysis highlighted the best SLH distance as the strongest predictor, explaining 43% of the variance in LEFT scores. These findings are consistent with those of prior studies that identified unilateral horizontal power as a key determinant of functional lower-limb performance [66,67]. Additional significant predictors included the standing long jump, countermovement jump, and VO_2peak._ Furthermore, recent studies have shown that CMJ, used as a proxy for peak power, is associated with the risk of injury [68,69]. However, research comparing countermovement jump force-time characteristics between athletes with a history of ACL injury and their uninjured counterparts, including female athletes, has reported no significant differences 11–13 months post-injury [70]. These findings differ from those observed in our study and may be attributed to the heterogeneity in the sample populations of previous studies, which included both male and female athletes. Additionally, variations in the methodologies used to assess injury risk may have contributed to inconsistent outcomes.

Furthermore, psychological resilience and GSES were modestly correlated with LEFT, indicating their potential utility in holistic athlete profiling. Specifically, GSES showed a significant predictive association with LEFT, suggesting that psychological factors could complement physical predictors in a comprehensive injury-risk assessment model. Previous research on self-efficacy supports these findings, highlighting the importance of psychological readiness and coping mechanisms in athletic performance and injury resilience [16,17,19]. Studies have highlighted the importance of addressing these psychological factors to enhance injury prevention and rehabilitation outcomes [57]. However, the limited variability in resilience scores in the current data may limit the ability to detect significant associations with LEFT, as study participants showed relatively high levels of resilience (mean score of 21/30), potentially leading to a ceiling effect. At the same time, the GSES showed a modest association with LEFT (r = −0.28). These findings should be interpreted with caution, and future studies are recommended to assess whether general self-efficacy mediates the relationship between resilience and LEFT using mediation analysis, to provide a clear understanding of the psychological mechanisms underlying the risk of athletic injury. The findings of the current study align with those of previous studies on multifactorial injury risk prediction across various sports. These studies indicate the need to incorporate functional performance testing, inter-limb asymmetry, and psychological factors into injury risk assessments. For instance, limb Y-Balance Test performance and functional asymmetries predicted sports injuries in competitive swimmers and volleyball players [71,72]. Similar findings were observed among CrossFit athletes and soccer players, in which psychological and training profiles, as well as previous injuries, correlated with injury prevalence and prevention [73].

When all predictors were considered simultaneously in the stepwise multivariate model, only SLH performance, VO_2peak_, and GSES remained as significant predictors. This finding suggests that these three variables independently explain a substantial proportion of the variance in LEFT outcomes, with the final model accounting for 68% of the total variance. The negative coefficients for SLH and VO_2peak_ indicate that greater hop distance and higher aerobic capacity were associated with faster LEFT completion times, which aligns with previous evidence linking lower limb power and cardiorespiratory fitness to multidirectional agility performance. The exclusion of other power and jump metrics (e.g., CMJ height, PP, MP) from the final model may reflect shared variance with SLH performance, highlighting the value of SLH distance as a robust field-based measure capturing multiple performance domains. These results underline the multifactorial nature of injury risk, demonstrating how both physiological and psychological characteristics integrate to influence athletic performance and safety. Incorporating these specific measures into routine pre-participation or in-season athletic assessments could enable the early identification of female athletes at heightened risk, thereby allowing for targeted, proactive intervention programs designed to mitigate injuries.

Despite this study’s strengths, it has several limitations. The cross-sectional design limited the ability to infer causation from the observed associations. A longitudinal design could provide insights into how these factors dynamically influence the risk of injury over time. Although the sample size met the a priori requirement, participants were recruited through convenience sampling from a single geographical region in Saudi Arabia, which may limit the generalizability of the findings. Importantly, cultural context and sports participation patterns may influence training exposure, competitive demands, injury reporting behaviors, and psychological readiness, potentially affecting the transferability of these findings to athletes from other regions or sports systems.

Additionally, the heterogeneous sample—comprising athletes from multiple sports, predominantly rugby and volleyball—further constrains sport-specific generalization. Thus, the LEFT assesses functional performance but does not directly measure injury incidence. Although the LEFT has demonstrated predictive validity in specific athletic populations, its sensitivity and specificity vary by sex and sport. In the present study, the heterogeneous sample comprising participants from multiple sports (predominantly rugby and volleyball) limited the generalizability of injury-related inferences. In the present study, the distribution of LEFT scores was highly unbalanced, with only four participants below the commonly used <135 s threshold [31,32]. While this approach allowed the examination of factors associated with poorer LEFT performance within a high-risk subgroup, it restricts score variability, limits sensitivity across the full performance spectrum, and reduces generalizability to lower-risk individuals. Therefore, LEFT findings should be interpreted cautiously as indicators of functional performance rather than direct measures of injury risk.

In addition, all participants in the present study were right-leg-dominant. As limb dominance can affect neuromuscular control and loading patterns, the exclusive inclusion of right-dominant individuals may limit the generalizability of the findings to left-dominant or mixed-dominance populations. Future research should include athletes with diverse limb dominance profiles and should better consider dominance-specific analyses to understand its role in functional performance and injury-related outcomes. Therefore, the results may be generalized with caution to comparable populations until further studies with larger sample sizes and multicenter longitudinal designs with direct injury tracking are conducted.

Therefore, future research should prioritize multicenter longitudinal designs with prospective injury surveillance, include athletes with diverse limb dominance profiles, and conduct sport-specific analyses to enhance sensitivity and external validity. Integrating functional performance measures with direct injury tracking across different cultural and sporting contexts will further clarify the role of combined physiological and psychological factors in preventing injuries.

## 5. Practical Applications

The findings of this study have several practical implications for clinicians, coaches, and sports performance practitioners working with female athletes. The identification of SLH performance, VO_2peak_, and self-efficacy as key independent predictors of functional performance suggests that these measures can be efficiently incorporated into routine screening and monitoring protocols. Functional field-based tests, such as the SLH and LEFT, combined with basic aerobic fitness assessments, provide a time- and cost-effective approach to identifying athletes at elevated risk of lower-extremity injury.

From a clinical perspective, these results support integrating functional performance testing and aerobic conditioning into injury prevention and return-to-sport decision-making, rather than relying solely on isolated strength measures. Additionally, the observed contribution of self-efficacy highlights the importance of incorporating psychological readiness into athlete evaluation. Targeted interventions aimed at enhancing confidence, motivation, and perceived ability to manage physical challenges may complement physical training strategies and further reduce susceptibility to injury.

## 6. Conclusions

This study highlights the combined influence of physiological and psychological factors on functional performance related to lower-extremity injury risk in female athletes. Using LEFT as a performance-based indicator, we identified SLH performance, VO_2peak_, and GSES as significant independent predictors of LEFT outcomes. Clinically, the mean LEFT completion time in this cohort (160 ± 22 s) exceeded the commonly reported normative thresholds (<135 s) [31,32], indicating a relatively higher baseline functional risk in this population. This underscores the importance of early functional screening in female athletes. In contrast, isokinetic muscle strength, anaerobic power, and psychological resilience did not significantly contribute to the final multivariate model results. These findings suggest that functional power, aerobic fitness, and self-efficacy play a more critical role in functional performance than isolated strength measures. From a practical perspective, these results support the use of targeted and efficient screening approaches. Incorporating SLH, aerobic capacity measures, and self-efficacy assessments into routine pre-participation or in-season evaluations may enable clinicians and coaches to identify athletes at an elevated risk and implement focused preventive interventions. Such an approach may be more time- and cost-efficient than comprehensive testing batteries that include fewer predictive measures.

## Figures and Tables

**Table 1:** Physical and training characteristics of participants (N = 60).

Variables	Mean	SD
Age (years)	24.50	6.90
Height (cm)	160.35	6.70
Weight (kg)	59.69	10.03
BMI (kg/m^2^)	23.12	3.14
Training time (day/week)	5	2
Sports experiences (months)	42	39
Duration of the training per day (min)	137	67
Type of sports, n (%) *		
Badminton, n (%) *	12	20
Basketball, n (%) *	8	13.3
Rugby, n (%) *	12	20
Football, n (%) *	6	10
Volleyball, n (%) *	16	26.7
Judo, n (%) *	6	10
Dominant leg, n (%) *		
Left, n (%) *	0	0
Right, n (%)	60	100

* Denotes data are presented as Frequency (n), and Percentage (%); Abbreviation: Standard Deviation (SD), Body Mass Index (BMI).

**Table 2:** Descriptive characteristics of mechanical and physiological variables assessed during the 30 s Wingate anaerobic test, isokinetic strength test, and cardiopulmonary exercise capacity.

Testing	Variable	Mean	SD
Wingate Anaerobic Test	Relative Anaerobic Peak Power (W/kg)	7.53	1.92
	Absolute Anaerobic Peak Power (W)	448.06	123.23
	Relative Mean Power (W/kg)	5.54	1.20
	Absolute Mean Power (W)	330	79.57
	FI (%)	60.63	15.41
Isokinetic Strength Test	PT extension (N·m)	184.55	44.60
	PT flexion (N·m)	95.08	21.44
	F:E ratio	53.50	14.08
CPET	VO_2peak_ (mL/kg/min)	40.82	5.8
	HR peak ((beats/min)	180	10
	VE (L/min)	82.06	17.31
	TV (L)	1.67	0.43
	Breathing Frequency (breaths/min)	50.63	9.46
	VE/VO_2ratio_	32.27	4.67
	VE/VCO_2ratio_	32.27	4.13
	PETO_2_ (mmHg)	105.88	4.70
	PETCO_2_ (mmHg)	31.46	3.93
	Peak Respiratory Exchange Ratio	1.10	0.06

Data are presented as mean ± Standard deviation (SD); Abbreviations: Watts (W); Fatigue Index (FI); Knee Flexion/Extension Strength Ratio (F:E); Peak Torque (PT), Newton (N); Cardiopulmonary exercise testing (CPET); Oxygen Consumption (VO_2_); Partial Pressure Of End-Tidal Carbon Dioxide (PETCO_2_); Partial Pressure Of End-Tidal Oxygen Dioxide (PETO_2_); Ventilatory Equivalent for Carbon Dioxide, (VE/VCO_2_) and Oxygen (VE/VO_2_); Minute Ventilation (VE); Tidal Volume (TV).

**Table 3:** Descriptive characteristics of injury risk parameters and self-efficacy and resilience score.

Variable	Mean	SD
LEFT (s)	160	22
Right SLH (cm)	132.13	23.56
Left SLH (cm)	129.73	25.03
Best SLH (cm)	134.41	24.21
SLH side-to-side difference (cm)	6.96	7.09
Standing Long Jump (cm)	156.61	26.94
CMJ Height (cm)	33.08	11
BRS (points)	21	4
GSES (points)	33	7.5

Data are presented as Mean ± Standard Deviation (SD) and Percentage (%). Abbreviations: Single-Leg Hop Test (SLH); Lower extremity functional test (LEFT); Peak Anaerobic Power (PAP); Countermovement Jump (CMJ); Watts (W); brief resilience scale (BRS); general self-efficacy (GSES); seconds (s).

**Table 4:** The associated *p*-values of the Pearson correlation coefficients.

Variables	Correlation Coefficients												
	1	2	3	4	5	6	7	8	9	10	11	12	13
LEFT (s)	1												
Best SLH (cm)	<0.001	1											
SLJ (cm)	<0.001	<0.001	1										
CMJ (cm)	<0.001	<0.001	<0.001	1									
PAP (W)	0.87	0.24	0.05	0.01	1								
Relative anaerobic PP (W/kg)	0.0005	0.001	0.0005	0.0003	0.96	1							
Relative AP (W/kg)	0.0004	0.001	0.0008	0.0003	0.93	<0.001	1						
VO_2peak_	<0.001	0.11	0.01	0.03	0.95	0.006	0.001	1					
PT extension (N·m)	0.67	0.31	0.26	0.96	<0.001	0.88	0.88	0.99	1				
PT flexion (N·m)	0.10	0.33	0.07	0.42	<0.001	0.12	0.24	0.37	0.0001	1			
F:E ratio	0.36	0.65	0.89	0.70	0.92	0.52	0.49	0.54	<0.001	0.003	1		
BRS	0.30	0.68	0.21	0.50	0.54	0.16	0.43	0.85	0.99	0.02	0.12	1	
GSES	0.02	0.48	0.60	0.67	0.75	0.77	0.68	0.99	0.91	0.34	0.46	0.001	1

Abbreviations: Single-Leg Hop Test (SLH); Lower extremity functional test (LEFT); Countermovement Jump (CMJ); Peak Power (W/kg); (AP) Average Peak Power, and Watts (W) Peak Torque (PT); Knee Flexion/Extension Strength Ratio (F:E); Brief Resilience Scale (BRS); General Self-Efficacy Scale (GSES).

**Table 5:** Univariate linear regression of association between LEFT as dependent variable and physiological and psychological variables.

Outcome Variable	Predictors	Coefficients						
		β	B	t	*p*-Value	95% Confidence Interval (CI)		Adj R^2^
						Lower	Upper	
LEFT	SLH	−0.66	−0.62	−6.83	<0.001	−0.80	−0.43	0.43
	SLJ	−0.55	−0.46	−5.06	<0.001	6.51	−0.64	−0.28
	CMJ Height	−0.51	−1.77	−4.60	<0.001	−2.54	−1.00	0.25
	PP	−0.43	−5.10	−3.68	0.001	−7.87	−2.32	0.17
	AP	−0.44	−8.28	−3.74	<0.001	−12.71	−3.85	0.18
	VO_2peak_	−13.81	−2.08	14.45	0.02	−25.59	−2.04	0.27
	GSES	−0.28	−0.85	−2.26	0.02	−1.60	−0.09	0.07

Significant level set at *p*-value < 0.05 of the whole model. Abbreviations: B: unstandardized beta “regression coefficient”; β: standardized beta; t: t-statistic test.

**Table 6:** Hierarchical regression analysis to the LEFT as dependent variable.

Model	Predictors	Coefficients ^a^							R^2^	∆R^2^	F
		B	β	SE	t	*p*-Value	95% CI				
							Lower	Upper			
Model 1 ^b^	Constant	-	273.58	16.18	16.91	<0.001	241.1	306.0	0.53	0.53	10.13 **
	SLH	−0.62	−0.58	0.15	−3.92	<0.001	−0.87	−0.28			
	SLJ	0.12	0.10	0.14	0.73	0.47	−0.17	0.37			
	CMJ	−01.0	−0.36	0.44	−0.83	0.41	−125	0.51			
	PP	−0.12	−1.42	3.29	−0.43	0.66	−8.03	5.18			
	AP	−0.02	−0.38	5.30	−0.07	0.94	−11.02	10.27			
	VO_2peak_	−0.23	−10.84	4.82	−2.25	0.02	−20.51	−1.16			
Model 2 ^c^	Constant	-	293.57	17.24	17.03	<0.001	258.9	328.16	0.59	0.05	6.62 *
	SLH	−0.58	−0.54	0.14	−3.85	<0.001	−0.83	−0.26			
	SLJ	0.12	0.10	0.13	0.83	0.41	−0.16	0.37			
	CMJ	−0.15	0.53	0.42	−1.26	0.21	−1.38	0.31			
	PP	−0.14	−1.68	3.13	−0.54	0.59	−7.98	4.61			
	AP	0.01	0.23	5.05	0.05	0.96	−9.90	10.37			
	VO_2peak_	−0.22	−10.32	4.58	−2.25	0.02	−19.53	−1.11			
	GSES	−0.23	−0.70	0.27	−2.57	0.01	−1.25	−0.15			

^a.^ Dependent Variable: LEFT; ^b.^ physiological outcome; ^c.^ Model 2 includes model 1 plus GSES. Abbreviations: B: unstandardized beta regression coefficient; β: standardized beta, SE: Standard errors, SLH: Single-Leg Hop Test; LEFT: Lower extremity functional test; CMJ: counter movement vertical jump; PP in W/kg: Peak Power; AP: Average Peak Power and GSES: General Self-Efficacy Scale; t: t-statistic test. Note: * denotes *p*-vlaue < 0.05; ** *p*-value < 0.001.

## Data Availability

Data are available for research purposes from the corresponding author upon reasonable requests. The individual de-identified participant data, statistical code, and additional materials supporting the findings of this study are available upon reasonable request from the corresponding author. The data are not publicly available due to privacy issue.

## Abbreviations

The following abbreviations are used in this manuscript:

AP	Average Power
BRS	Brief Resilience Scale
CMJ	Counter Movement Vertical Jump
GSES	General Self-Efficacy
FI	Fatigue Index
LEFT	Lower-Extremity Functional Test
PT	Peak Torque
PAP	Peak Anaerobic Power
PP	Peak Power
SLH	Single-Leg Hop
SLJ	Standing Long Jump
VO_2peak_	Peak Oxygen Uptake
W	Watts
